# Effective strategies for typhoid conjugate vaccine delivery: Health and economic insights from the 2015 Kampala outbreak

**DOI:** 10.1371/journal.pntd.0013566

**Published:** 2025-10-07

**Authors:** Yeonsu Lee, Pamela Kim N. Salonga, Changdae Son, Geunsoo Jang, Dae-Hyup Koh, Jong-Hoon Kim, Hyojung Lee

**Affiliations:** 1 Department of Statistics, Kyungpook National University, Daegu, Republic of Korea; 2 School of Computing and Augmented Intelligence, Arizona State University, Tempe, Arizona, United States of America; 3 Epidemiology, Public Health, Impact, International Vaccine Institute, Seoul, Republic of Korea; Public Health Agency of Canada, CANADA

## Abstract

Typhoid fever remains a major public health threat in low- and middle-income countries (LMICs), where inadequate access to clean water and sanitation drives recurrent outbreaks. With antimicrobial resistance on the rise, the urgency of deploying preventive strategies such as typhoid conjugate vaccines (TCVs) have grown. In this study, we developed a dynamic compartmental model calibrated to the 2015 typhoid outbreak in Kampala, Uganda, to assess the health and economic outcomes of various outbreak response immunization (ORI) strategies using TCVs. We aimed to identify optimal ORI strategies that minimize cases and typhoid-related deaths as well as the costs of implementation. Our model incorporated different phases of the outbreak, vaccine coverage levels (30%, 50%, 70%), timing (early, late, combined), and campaign duration. Cost-effectiveness was evaluated based on disability-adjusted life years (DALYs) and incremental cost-effectiveness ratios (ICERs), using World Health Organization (WHO) thresholds derived from Uganda’s 2015 gross national income per capita. Early, high-coverage vaccination (Scenario 1) was most impactful reducing the effective reproduction number (*R*_t_) below 1 during the epidemic peak and averting over 7,000 cases including 180 deaths. The timing of vaccine deployment was the most critical determinant of effectiveness, followed by coverage level and campaign duration. Our findings highlight the importance of rapid, high-coverage TCV deployment at the early stages of an outbreak. Strengthening disease surveillance and improving vaccine logistics are essential for a timely response. This modeling framework offers actionable evidence to support policy development and optimize outbreak preparedness in typhoid-endemic regions.

## Author summary

Typhoid fever remains a serious public health concern in countries with limited access to safe water and sanitation. Increasing resistance to treatment highlights the need for preventive measures such as early vaccination. Using a mathematical model of the 2015 Kampala outbreak, this study explored the effective strategy of timing, duration, and coverage of vaccination campaigns. We found that rapidly vaccinating many people at the beginning of a Typhoid fever outbreak can prevent thousands of infections and hundreds of deaths, while also lowering the overall costs associated with typhoid. In contrast, delayed or limited vaccination greatly reduces these benefits. These findings emphasize that timely and broad deployment of typhoid conjugate vaccines is the most effective and cost-efficient strategy. Strengthening surveillance systems and ensuring efficient vaccine delivery are key to improving overall outcomes. This evidence provides valuable guidance for policymakers and health authorities in designing outbreak response strategies to reduce typhoid burden.

## 1. Introduction

Typhoid fever, caused by the bacterium Salmonella enterica serovar Typhi (S. Typhi), remains a significant global public health concern, particularly in low- and middle-income countries (LMICs) where access to clean water, sanitation, and reliable healthcare infrastructure is limited [1]. This bacterial infection is transmitted through the ingestion of contaminated food or water and is characterized by symptoms such as high fever, abdominal pain, and gastrointestinal disturbances. Without appropriate and timely treatment, typhoid fever can lead to severe complications and even death. Despite advancements in sanitation and healthcare, typhoid fever continues to pose a substantial burden on affected communities, highlighting the urgent need for effective prevention and control measures [2–5]. As of 2019, the World Health Organization (WHO) reports that an estimated 9 million people suffer from typhoid infection every year, among which 110,000 individuals die of typhoid-associated deaths [6].

Because of the nonspecific manifestations of typhoid disease, accurate diagnosis is critical to guarantee that infected individuals receive treatment on time. However, the shortage of rapid and accurate diagnostic tools remains an important challenge in controlling typhoid fever outbreaks. This is further exacerbated by the existence of chronic carriers, who are known to be asymptomatically infected individuals and able to transmit the infection for an extended period of time. Improvements in sanitation combined with the development of antibiotics have been valuable in mitigating further transmission and managing morbidity in infected individuals. However, due to the increasing antimicrobial resistance, more effective intervention strategies are needed. Two vaccines, the Vi polysaccharide vaccine and the Ty21a vaccine, have been in use as prevention for at-risk populations. However, these vaccines do not provide long-lasting protection and are not safe for children aged 2 years and below. This poses a challenge as children aged 14 years and below constitute a considerable portion of the typhoid fever burden [1,2,4]. Moreover, these vaccines are generally recommended for travelers and not widely integrated into routine immunization programs, which limits their public health impact [7].

In recent years, the development and introduction of typhoid conjugate vaccines (TCVs) have offered new hope in the fight against typhoid fever. As of February 2024, the WHO has prequalified three typhoid conjugate vaccines: Typbar-TCV (Bharat Biotech International Limited), TYPHIBEV (Biological E. Limited), and SKYTyphoid (SK Bioscience Co., Ltd) [8,9]. Unlike previous vaccines, TCVs provide longer-lasting protection and can be administered to children as young as six months old, making them particularly suitable for use in endemic regions [10–12]. By targeting the outer membrane polysaccharide of S. Typhi, TCVs stimulate a robust immune response, effectively preventing infection and reducing transmission within communities. TCVs have been introduced into nationwide routine immunization programs, starting from Navi Mumbai, India in 2018 [13]. Subsequently, Pakistan (2019–2022) [14], Liberia (2021) [15], Zimbabwe (2021) [16], Nepal (2022) [17], Samoa (2021–2022) [18], and Malawi (2023) [19] have implemented TCV introduction campaigns. This has resulted in over 59 million people being vaccinated as of 2022 [20].

The potential of TCVs to mitigate the impact of typhoid fever outbreaks holds promise for improving public health outcomes, especially in resource-limited settings where the disease burden is most significant. Despite the growing recognition of TCVs as a valuable tool in typhoid fever prevention, their use in outbreak response scenarios remains underexplored. The TCV has only been used in response to outbreaks in Pakistan and Zimbabwe. In 2018, Hyderabad, Pakistan [11] initiated a TCV campaign to vaccinate 207,000 children aged 6 months to 10 years in response to an extensively drug-resistant typhoid fever outbreak. Following this, in 2019, Karachi [21] vaccinated 87,993 children aged 6 months to 15 years. In 2019, Harare, Zimbabwe conducted a TCV campaign to vaccinate 318,698 high-risk populations to control the outbreak [22]. In these cases, large-scale immunization campaigns targeted children and high-risk populations to curb escalating outbreaks, but broader evaluation of their effectiveness and cost-efficiency is lacking.

In light of the recent COVID-19 pandemic, mathematical modeling has emerged as a powerful approach for analyzing infectious disease dynamics and informing public health decisions, especially when timely field data are limited. Models can simulate various scenarios, assess the impact of interventions, and forecast outcomes under different assumptions. Several mathematical models of typhoid dynamics exist in the literature [23–30]. In Pitzer et al. [27], a simplified typhoid fever transmission model considering both long- and short-cycle transmission was developed to assess the potential impacts of vaccination in the endemic region of South Asia. Antillon et al. [28] modified this model by considering only the short-cycle transmission and assessed the cost-effectiveness of TCV vaccination in LMICs. In [30], Saul et al., presented a stochastic agent-based model to simulate the transmission of typhoid, emphasizing the significance of long-lasting immunity and the carrier state in managing typhoid fever, especially for optimizing control strategies like vaccination. Analyses of how these various models demonstrate the cost-effectiveness of routine vaccination with TCV in most settings are also explored in [31–33]. In outbreak response modeling, historical data and mathematical models are used to simulate counterfactual scenarios to estimate possible outcomes had different responses been implemented during the outbreak [34]. Findings in these studies can be useful in guiding decision-making in future outbreaks. Particularly, assessing the impact of outbreak response immunization (ORI) programs using mathematical models can help policymakers and program implementers identify elements of the response that are most critical to either prevent or at least minimize the scale of an outbreak [35]. In regions like Uganda, where typhoid fever outbreaks periodically occur, the implementation of timely and effective vaccination strategies could significantly reduce morbidity and mortality associated with the disease. However, the absence of empirical studies evaluating the feasibility and impact of TCV deployment during outbreaks leaves a critical gap in our understanding of their potential benefits.

Cost-effectiveness analyses using the Incremental Cost-Effectiveness Ratio (ICER), which measures the cost per disability-adjusted life year (DALY) averted, further highlight the value of TCV deployment. Studies have demonstrated that TCV introduction yields substantial reductions in both disease burden and healthcare costs, particularly in settings where typhoid fever remains a high-risk disease [28,31]. The ICER calculation for routine TCV vaccination often shows that it is a cost-effective intervention, offering significant health benefits at a reasonable cost, especially in regions with high disease incidence and limited access to healthcare [28,32]. In outbreak scenarios, where the timely deployment of TCV can prevent the spread of the disease, these vaccines prove to be even more cost-effective, reducing not only the incidence of typhoid but also the economic strain of outbreak management [32,33].

In the present study, we developed a dynamic compartmental model calibrated to the 2015 typhoid outbreak in Kampala, Uganda. This work evaluates the health and economic impacts of varying ORI strategies considering variations in the timing, coverage, and duration of vaccination campaigns. Our goal is to inform evidence-based-ORI policies that optimize both public health and economic outcomes in resource-constrained, typhoid-endemic regions. By leveraging real outbreak data and rigorous modeling, this research provides novel insights into the strategic use of TCVs for outbreak response.

## 2. Materials and methods

### 2.1. Data description

We utilized the daily incidence data from the January 1-June 12 2015 outbreak in Kampala, Uganda, consisting of 10,230 suspected cases defined by clinical symptoms such as fever, abdominal pain, and headache [36]. Because time series data were not available in the original outbreak report, we extracted 163 data points from Fig 2 of the article by Kabwama et al. using WebPlotDigitizer. The extraction process was validated by comparing the digitized time series data with original time series data in reports where both the raw data and corresponding plots are available. More details are described elsewhere [37]. Although, the data provides no information on disease-induced mortality or complications such as perforations, they capture key temporal patterns of the outbreak, which are essential for model calibration (see Section 2.2 for details).

**Fig 1:**
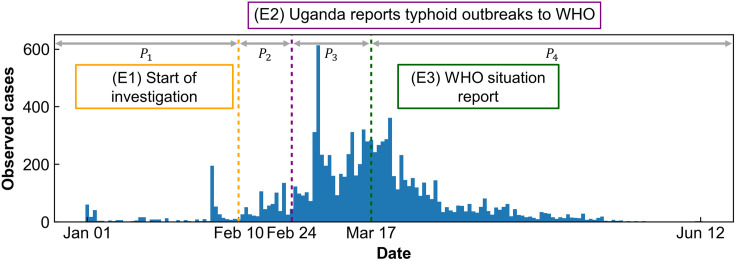
The number of observed cases in the Kampala region of Uganda. Epidemic curve of the typhoid outbreak in Kampala, Uganda, from January 1 to June 12, 2015. Based on three critical time points, (E1)–(E3) related to the outbreak (indicated by yellow, purple, and green dotted lines), the timeline was divided into four distinct periods for application in the mathematical model: *P*_*1*_ (January 1–February 10), *P*_*2*_ (February 10–February 24), *P*_*3*_ (February 24–March 17), and *P*_*4*_ (March 17–June 12).

These following critical time points were incorporated into the mathematical modeling framework to ensure temporal alignment with observed epidemiological events.

(E1) **Start of investigation**: A typhoid-related death was first reported, prompting the Ugandan government to launch an official investigation on February 10, 2015 [36].

(E2) **Uganda reports typhoid outbreaks to WHO**: The outbreak was formally reported to the World Health Organization (WHO) on February 24, 2015 [38].

(E3) **WHO situation report**: The WHO subsequently issued an official situation report on March 17, 2015 [38].

To reflect the progression of the outbreak and corresponding public health responses, we divided the epidemic timeline into four distinct phases: *P*_*1*_ (January 1–February 10, 2015), *P*_*2*_ (February 10–February 24, 2015), *P*_*3*_ (February 24–March 17, 2015), and *P*_*4*_ (March 17, 2015–June 12, 2015). These intervals were utilized to parameterize the model dynamically and assess the impact of interventions across different stages of the outbreak.

### 2.2. Definition of cases and population

There are natural delays in outbreak detection and response, blood culture testing is not always readily accessible to identify confirmed cases, especially for neglected tropical diseases such as typhoid fever [22,37]. Thus, the time series of suspected cases might provide better information on the trajectory of an outbreak instead of the number of confirmed or probable cases. Hence, in this work, the time series of recorded suspected case data is used to validate the mathematical model. We refer to the suspected cases as observed cases for the rest of the paper.

For our data analysis, we used the population of Kampala as reported by Kabwama et al. (2017), which was estimated at 1.4 million (1,400,000). Kampala has five divisions: Kampala Central, Kawempe, Makindye, Rubaga, and Nakawa. The epidemiologic investigation focused on two markets and a commuter taxi park in Kampala Central Division, where the initial cases were concentrated. Including this geographic information clarifies the spatial focus of the outbreak and the scope of the data used in the analysis [36]. This population estimate accounts for the urban density and demographic structure relevant to typhoid transmission and was used as the baseline for model calculations. Fig 1 illustrates the epidemic curve of the 2015 Kampala outbreak.

### 2.3. Model description

**Fig 2:**
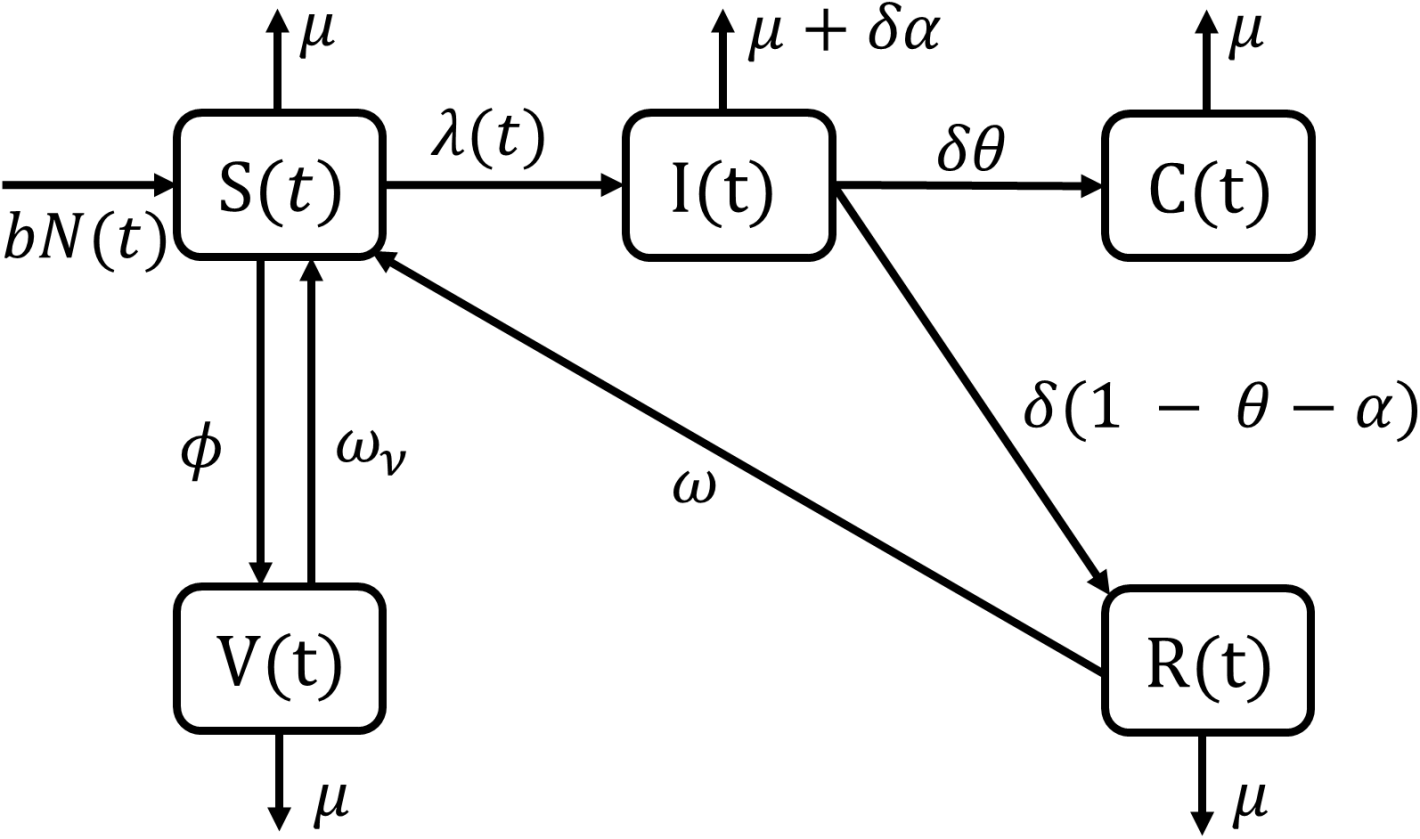
Compartmental model for typhoid transmission in an endemic setting. The compartments are composed of susceptible *S*(*t*), infections *I*(*t*), recovered *R*(*t*), chronic carriers *C*(*t*), and vaccinated *V*(*t*).

Our typhoid transmission model consists of five compartments: the susceptible population *S*(*t*), infectious *I*(*t*), recovered with temporary immunity *R*(*t*), chronic life-long carriers *C*(*t*), as well as vaccinated individuals *V*(*t*) derived from the susceptible population. We denote the total population at time *t* as the sum of individuals in all compartments, N(t)=S(t)+I(t)+R(t)+C(t)+V(t). The parameters used in the model are summarized in Table 1. Our model, illustrated in Fig 2, is governed by the system of ordinary differential equations given in Eq. (1).

**Table 1:** Parameter values of the typhoid model.

Parameter	Description	Value	Reference
*b*	Birth rate	0.039/year	[39]
*μ*	Natural death rate	0.007/year	[39]
*β*(*t*)	Transmission rate at time *t*	–	Estimated
*f*	Reporting rate	0.2	Assumed
*γ*	Relative infectiousness of chronic carriers	0.0009	Assumed
*κ*	Vaccine coverage	0	Assumed (30%, 50%, 70%)
v	Vaccine efficacy	0.8	[12]
1/δ	Duration of infectiousness	11.8 days	[40]
*θ*	Probability of becoming a Chronic carrier	0.029	[41]
*α*	Disease-induced mortality	0.025	[42]
1/ω	Duration of natural immunity	2 years	[43]
$\text{1}/\omega_{\nu }$	Duration of vaccine-induced immunity	4 years	[12]


$$\frac{dS(t)}{dt}=bN(t)-\lambda (t)S(t)-\phi +\omega_{\;\nu }V(t)-\mu S(t)$$

$$\frac{dI(t)}{dt}=\lambda (t)S(t)-\delta I(t)-\mu I(t)$$

$$\begin{array}{c} \frac{dR(t)}{dt}=\delta (\text{1}-\theta -\alpha )I(t)-\omega R(t)-\mu R(t)\; \end{array}$$ (1)

$$\frac{dC(t)}{dt}=\delta \theta I(t)-\mu C(t)$$

$$\frac{dV(t)}{dt}=\phi -\omega_{\nu }V(t)-\mu V(t)$$

$$\lambda (t)=\frac{\beta (t)}{N(t)}\left(I(t)+\gamma C(t)\right)$$

There are several assumptions for the typhoid model. All individuals are born completely susceptible to typhoid infection at the rate *bN*(*t*), where *b* is the birth rate and *N*(*t*) is the total population size at time *t*. Upon ingestion of contaminated food or water, also referred to as direct or short-cycle transmission, a susceptible individual *S*(*t*) becomes infected at *λ*(*t*), also known as force of infection. Here, we assume that infected compartments *I*(*t*) drives disease transmission, with chronically infected individuals *C*(*t*) contributing to a lesser extent, scaled by a factor 0<γ<1. Since typhoid infection is self-limiting, most infected individuals naturally recover and gain temporary immunity to reinfection (*ω*). Moreover, although not explicitly included in the model, some individuals get better by taking antibiotics and subsequently move to the recovered compartment *R*(*t*). Nevertheless, a fraction *α* die from severe complications, and a frac*t*ion *θ* become life-long chronic carriers. Since this is an endemic model, we assume individuals can die of natural causes and leave their respective compartments at the rate *μ*.

We assume that for a given vaccine coverage, the total amount of vaccine administered is constant, calculated as $\kappa \nu N_{\text{0}}$ where *κ* represents vaccine coverage, v represents vaccine efficacy and *N*_0_ represents the total population, assumed to be 1.4 million. ϕ refers to the weekly number of vaccinated individuals, which is defined by $\phi =\frac{\kappa \nu N_{\text{0}}}{T}$, where *T* denotes the vaccination period. In the model, vaccination is represented as a transition from the susceptible compartment *S*(*t*) to the vaccinated compartment *V*(*t*). The vaccine-induced protection is also temporary; thus, individuals in vaccinated compartments become susceptible again at a ra*t*e $\omega_{\nu }$. Due to lack of information, we assumed that there is no vertical transmission. Moreover, we also did not explicitly model indirect or long-cycle transmission through contamination of the broader environment such as pollution of waterways by human feces. The initial values for each compartment are summarized in Table A in S1 Appendix.

### 2.4. Reproduction number

Following the model description, we define the basic reproduction number (*R*_0_) to estimate the transmission rate (*β*), and the effective reproduction number (*R*_t_) as key indicators of transmission potential. The basic reproduction number *R*_0_ represents the average number of secondary infections caused by a single infectious individual in a fully susceptible population. The effective reproduction number *R*_t_ reflects the effective transmission potential at a given time *t*, accounting for depletion of susceptible individuals within compartments and the temporal variation in the transmission rate driven by specific events.

Both *R*_0_ and *R*_t_ were derived using the next generation matrix approach, as detailed in Section A in S1 Appendix. Based on this method, the expression for *R*_0_ was obtained as Eq. (2).


$$\begin{array}{c} R_{\text{0}}=\frac{\beta (t)(\mu +\delta \theta \gamma )}{\mu (\delta +\mu )}\; \end{array}$$ (2)

The basic reproduction number *R*_0_ consists of $R_{\text{0},\text{I}}=\frac{\beta (t)\mu }{\mu (\delta +\mu )}$ contributed by *I*(*t*) and $R_{\text{0},\text{C}}=\frac{\beta (t)\delta \theta \gamma }{\mu (\delta +\mu )}$ contributed by *C*(*t*), respec*t*ively (i.e., $R_{\text{0}}=R_{\text{0},\text{I}}+R_{\text{0},\text{C}}$). The proportion $R_{\text{0},\text{C}}/R_{\text{0}}$ refers to the proportion of *R*_0_ attribu*t*able to chronic carriers, defined as $R_{\text{0},\text{C}}/R_{\text{0}}=\frac{\delta \theta \gamma }{\mu +\delta \theta \gamma }.$

Further accounting for the changes in the number of individuals within compartments and the temporal variation in the transmission rate, the effective number was given as Eq. (3).


$$\begin{array}{c} R_{\text{t}}=\frac{\beta (t)S(t)(\mu +\delta \theta \gamma )}{N(t)\mu (\delta +\mu )}\; \end{array}$$ (3)

Here, the transmission rate is modeled as a time-dependent parameter *β*(*t*) because transmission potential can vary in practice as a result of specific events. As a threshold condition, values of *R*_0_ or *R*_t_ greater than 1 imply sustained transmission in the population, in contrast, values below 1 indicate that the outbreak is not self-sustaining [44,45].

In addition, we calculated the parameter *γ*, which refers to relative infectiousness of chronic carriers. Following Pitzer et al. [27], we calculated the proportion of transmission attributable to carriers based on *R*_0_. Based on this approach, *γ* was calculated by assuming *R*_0_ as 2.49 [27] and that infections caused by chronic carriers account for 10% of the total infections (i.e., $R_{\text{0},\text{C}}/R_{\text{0}}=\frac{\delta \theta \gamma }{\mu +\delta \theta \gamma }=\text{0}.\text{1})$.

### 2.5. Parameter estimation

The time period from January 1, 2015 to June 12, 2015 is divided into four intervals, such as *P*_*1*_ (January 1–February 10), *P*_*2*_ (February 10–February 24), *P*_*3*_ (February 24–March 17), and *P*_*4*_ (March 17–June 12). We defined *incidence*(*t*) as fI(t), where *f* denotes the reporting rate. Considering the underreporting observed in sub-Saharan Africa, we assume that a proportion *f* of the model-predicted new infections corresponds to the observed data [27,28]. The cumulative cases are calculated by sum of *incidence*(*t*).

For each time interval *P*_*i*_, we estimate the transmission rate *β*_*i*_ by minimizing the loss function as $(incidence(t)-obs(t){)}^{\text{2}}$ by using the least-squares method, where *obs*(*t*) denotes the observed data shown in Fig 1. For *P*_*1*_, we assumed *R*_0_ = 2.49 [27] and calcula*t*ed *β_1_* = 0.19, rather than estimated directly from data. Based on the definition of *R*_0_ obtained through the next generation matrix approach *β_1_* was computed as Eq. (4).


$$\begin{array}{c} \begin{array}{c} \beta_{\text{1}}=R_{\text{0}}\frac{\mu (\delta +\mu )}{\mu +\delta \theta \gamma }\; \end{array} \end{array}$$ (4)

While *P*_*2*_, *P*_*3*_, and *P*_*4*_, the values of *β_i_* were estimated through the data fitting. In other words, ${\beta (t=\;\{\beta }_{i}\}$ is the transmission rate defined in the model. *R*_t_ is the effective reproduction number, and for each time interval *P*_*i*_, we compute the average value of *R*_t_, denoted as *E*(*R*_t_).

### 2.6. Analysis of vaccination strategies against typhoid infection

Historically, mathematical models have been proven to be useful in analyzing disease outbreaks and informing outbreak response and health policy in the real world. In practice, a baseline scenario is first constructed by fitting the model to the historical data. Then, counterfactual scenarios, such as no-vaccination and various levels of vaccination response, are considered to quantify how each strategy could have impacted the dynamics of the outbreak. Ultimately, this analysis aims to provide an evidence-based recommendation for the selection of efficient and optimal measures for future outbreaks. In this work, to compare different ORI strategies, we adopt part of the framework introduced by Delport et al. [35], where they identified three main metrics to assess the impact of ORI programs. These metrics are health impacts, economic impacts, and the risk of severe outbreaks. In this work, in particular, we aim to measure the health and economic impact of various ORI strategies.

Mathematical models have demonstrated that vaccination is among the most cost-effective outbreak response strategies for vaccine-preventable diseases. In particular, timely vaccination has the potential to significantly reduce both morbidity and mortality. These models can inform vaccine response strategies in various ways, including by highlighting the importance of timely immunization, identifying high-risk areas, prioritizing the allocation of limited vaccine resources, detecting and addressing surveillance gaps, and evaluating both short- and long-term benefits [46]. In this study, we focus specifically on the short-term effects of vaccination and the importance of timely implementation to ensure cost-effective outbreak interventions.

We assess the health impact by measuring the corresponding case reduction rate and *R*_t_. The case reduction rate was computed as the relative decrease in cumulative cases under each vaccination scenario compared to the no-vaccination baseline. Here, *C*_*no vacc*_ denotes the cumulative number of cases in the no-vaccination scenario, and *C*_*vacc*_ denotes the cumulative number of cases under a given vaccination scenario. The formula is as follows:


$$\begin{array}{c} Reduction\;rate\;=\;\frac{C_{no\;vacc}-\;C_{vacc}}{C_{no\;vacc}}\times \text{1}\text{0}\text{0}\% \end{array}$$ (5)

On the other hand, we quantify the economic impact by measuring the incremental cost-effectiveness ratio relative to the disability-adjusted life years averted and to cases averted, respectively. These metrics provide a quantitative framework for assessing the potential impact of different vaccination strategies under realistic outbreak conditions. In addition, according to Delport et al. [35], three elements of response strategies can be varied by program administrators to maximize the positive impact of a response: scale of response, speed of response, and prioritization of delivery. Varying the prioritization of delivery implies targeting the ORI programs among vulnerable groups or contacts of known cases. Since our model is not age-stratified, this metric is not applicable in this study. Hence, we only consider various strategies that account for changes in the scale of the response by varying the vaccine coverage and changes in the speed of the response by varying the timing and duration of vaccine deployment.

Each strategy considered in the analysis is represented by two varying aspects of ORI deployment: vaccine coverage and vaccine timing. We vary the vaccine coverage from 30% to 70%, with 20% increments. On the other hand, we consider three vaccine timing strategies which are described in Table 2. The earliest possible starting point for vaccination was set as February 10, 2015, the date when the official investigation of suspected typhoid cases began. Subsequently, the vaccination period was determined by considering realistic constraints such as feasible initiation time and campaign duration. This allowed for a practical exploration of how different levels of vaccination coverage influence disease dynamics.

**Table 2:** Strategy comparisons for deploying typhoid conjugate vaccines.

Scenario	Vaccination strategy
Scenario 1: Early	Vaccination during *P*_*2*_ (February 10–February 24, 2015)
Scenario 2: Late	Vaccination during *P*_*3*_ (February 24–March 17, 2015)
Scenario 3: Combined	Vaccination during *P*_*2*_ + *P*_*3*_ (February 10–March 17, 2015)

### 2.7. Cost-effectiveness analysis

The cost-effectiveness analysis presented in this work evaluates the impact of various typhoid vaccination strategies implemented at different time periods during the 2015 Kampala outbreak. The analysis follows a decision-analytic framework, comparing health and economic outcomes under different scenarios of vaccination timing and coverage against a no-vaccination baseline.

Health outcomes of this cost-effectiveness analysis include total and averted typhoid cases and deaths, and Disability-Adjusted Life Years (DALYs) averted. DALYs provide a composite measure of disease burden, capturing both premature mortality and morbidity. They are defined as the sum of Years of Life Lost (YLL) due to premature death and Years Lived with Disability (YLD) from illness, i.e., DALY = YLL + YLD. To compute DALYs, the analysis incorporated age-stratified typhoid incidence, case fatality rates, disability durations, and life expectancy.

Economic outcomes, on the other hand, were estimated based on vaccination expenses and treatment costs for inpatient and outpatient care [10,47]. Vaccination costs were computed as a sum of three components: US$1.50 for vaccine procurement, US$0.24 for injection and safety equipment, and US$1.76 for delivery, based on Ugandan estimates reported in Bilcke et al. [31]. In the same paper [31], treatment costs were valued as US$43 for inpatient care and US$1.4 for outpatient care.

The main economic outcome measure was the Incremental Cost-Effectiveness Ratio (ICER), calculated as total cost divided by either DALYs averted or cases averted as in Eq. (6).


$$\begin{array}{c} ICER=\frac{Vaccination\;Cost\;+\;Treatment\;Cost}{DALYs\;Averted\;or\;Cases\;Averted} \end{array}$$ (6)

To assess the economic efficiency of each intervention, ICERs were compared against established cost-effectiveness thresholds. Interventions were classified as highly cost-effective (HCE) if the cost per DALY averted was less than or equal to US$656.60, and cost-effective (CE) if less than or equal to US$1,969.80. These thresholds are commonly used in global health economics and are derived from per capita gross domestic product (GDP) benchmarks in Uganda in 2015, aligning with WHO guidelines [48,49]. Table 3 summarizes the parameters related to the cost-effectiveness analysis.

**Table 3:** Parameters related to the cost-effectiveness analysis.

Parameter	0-14 years	15-59 years	60 + years	Reference
Proportion of cases by age groups	9.4%	90.1%	0.5%	[36,50]
Representative age	0	30	60	assumed
Remaining life expectancy	61 years	45 years	15 years	[51]
Case fatality rate (CFR)	0.025			[42]
Duration of disability	16 days			[31]
Disability weights	0.053			[31]
Inpatient treatment cost per person	US$43			[31]
Outpatient treatment cost per person	US$1.4			[31]
Vaccine procurement cost per dose	US$1.5			[31]
Vaccine safety and injection cost per dose	US$0.24			[31]
Vaccination delivery cost per dose	US$1.76			[31]

For clarity, the cost-effectiveness study provides the following definitions by key terms:

(i) Vaccination Cost refers to the total expense associated with getting a vaccine administered to all covered individuals. It is calculated as the product of the total number of individuals to be vaccinated and the vaccination cost per dose as follows: Vaccination cost = (Vaccine procurement cost per dose + Vaccine safety and injection equipment cost per dose + Vaccination delivery cost per dose) $\times N_{\text{0}}\times \kappa$.
(ii) Treatment Cost refers to the total financial resources required to manage patients with the disease. It is calculated as the product of the cumulative number of cases and the per capita treatment cost as follows: Treatment Cost = (Inpatient treatment cost per person + Outpatient treatment cost per person) ×f× Cumulative cases.
(iii) DALYs averted refers to the number of healthy life years preserved due to an intervention, estimated as the difference between the DALYs in a no-intervention scenario (*DALY*_*no vacc*_) and the DALYs in an intervention scenario (*DALY*_*vacc*_) [52].
(iv) Cases averted refers to the cumulative number of cases prevented due to an intervention, computed as the difference between the cumulative number of cases without the intervention (*C*_*no vacc*_) and the expected number of cases with the intervention (*C*_*vacc*_) (i.e., Cases averted = $\;C_{no\;vacc}-C_{vacc}$).
(v) YLL refers to the years of life lost from dying prematurely (calculated using Eq. (7)), while YLD measures the healthy years of life lost due to living with the disability caused by the disease (calculated using Eq. (8)). These are calculated as follows [53]:

$$\begin{array}{c} \text{YLD}=\text{Cumulative cases}\times (\text{1}-\text{CFR})\times \text{Duration of disability}\times \text{Disability weights } \end{array}$$ (7)

$$\begin{array}{c} \text{YLL}=\text{Cumulative cases}\times \text{CFR}\times \text{Remaning life expectancy } \end{array}$$ (8)

Three scenarios modeled vaccine coverage levels (70%, 50%, and 30%) introduced at different outbreak phases, early (*P*_*2*_), late (*P*_*3*_), and combined (*P*_*2* _+ *P*_*3*_), allowing for the assessment of both the timing and intensity of vaccine rollout on health and economic efficiency.

## 3. Results

### 3.1. Simulation results for vaccine scenarios

**Fig 3:**
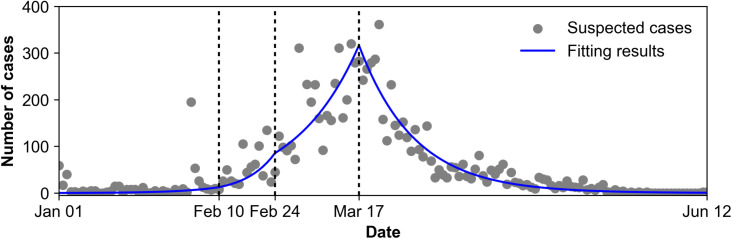
Fitting results of the epidemic model without vaccination. The gray dots represent the number of cases in Kampala, Uganda in 2015, and the blue line shows the fitting results using the epidemic model without vaccination. The simulation period is divided into one period before the investigation (*P*_*1*_) and three periods (*P*_*2*_–*P*_*4*_) for model fitting.

Here, we present our simulation results of the impact of ORI using TCVs with *R*_t_ and case reduction rate relative to the no vaccination scenario. As a preliminary step, we performed model simulation using *R*_0_ during the *P*_*1*_ period, followed by model fitting to the observed data in each of the predefined periods (*P*_*2*_– *P*_*4*_) as described in the previous section, in order to estimate the baseline epidemic trend in the absence of vaccination (Fig 3). Table 4 presents the interval-based fitting results of the mathematical model without considering vaccination. These results will be used as a basis for comparison with the outcomes of the vaccination simulation.

**Table 4:** Estimated *β*_*i*_ and reproduction numbers for each time period, $P_{\text{1}}-P_{\text{4}}$.

Symbol	Period			
	*P_1_*	*P_2_*	*P_3_*	*P_4_*
*β*_*i*_	*β*_*1*_ = 0.19	*β*_*2*_ = 0.22	*β*_*3*_ = 0.14	*β*_*4*_ = 0.01
Reproduction number	*R*_0_ = 2.49	*E*(*R*_t_) = 2.87	*E*(*R*_t_) = 1.86	*E*(*R*_t_) = 0.15

**Fig 4:**
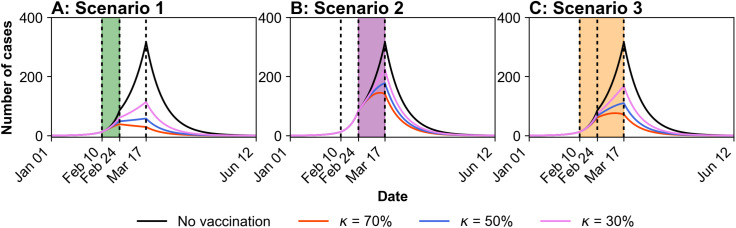
Model simulations under different vaccination scenarios. These are the simulation results for three scenarios, with each scenario varying by vaccination start time and duration for three vaccine coverage levels (κ). Vaccination is implemented only during the highlighted periods. **A.** The vaccination period corresponds to *P*_*2*_ (February 10–February 24, 2015). **B.** The vaccination period corresponds to *P*_*3*_ (February 24–March 17, 2015). **C.** The vaccination period includes both *P*_*2*_ and *P*_*3*_ (February 10–March 17, 2015).

Fig 4 presents the results of different vaccination strategies. Panels A, B, and C display the simulated daily number of cases under three distinct scenarios, each corresponding to a specific vaccination coverage strategy. In each panel, each line color represents a fixed vaccine coverage, and the respective values assigned are listed in the Fig legend. To facilitate a comparative analysis of vaccine effectiveness, the estimated values of *R*_t_ for each period, along with the corresponding reduction rates in cumulative case counts calculated using Eq. (5) for each period, were summarized in Table 5. The no-vaccination scenario was used as the baseline for comparison.

**Table 5:** Estimated effective reproduction numbers (*R*_t_) and cumulative case counts for each vaccination scenario.

Vaccine coverage (%)	Period	*E*(*R*_t_)				Cumulative case counts			
		No vaccination	Scenario 1	Scenario 2	Scenario 3	No vaccination	Scenario 1	Scenario 2	Scenario 3
κ = 70%	*P*_*2*_	2.87	2.14	2.87	2.57	38.78	26.29 (-32.2%)	38.78 (-0.0%)	32.7 (-15.7%)
	*P*_*3*_	1.86	0.9	1.39	1.18	177.66	33.6 (-81.1%)	126.36 (-28.9%)	71.83 (-59.6%)
	*P*_*4*_	0.15	0.07	0.07	0.07	51.59	4.43 (-91.4%)	21.3 (-58.7%)	10.86 (-78.9%)
κ = 50%	*P*_*2*_	2.87	2.35	2.87	2.66	38.78	29.17 (-24.8%)	38.78 (-0.0%)	34.29 (-11.6%)
	*P*_*3*_	1.86	1.17	1.52	1.37	177.66	52.85 (-70.3%)	138.34 (-22.1%)	91.9 (-48.3%)
	*P*_*4*_	0.15	0.09	0.09	0.09	51.59	8.93 (-82.7%)	27.41 (-46.9%)	16.95 (-67.1%)
κ = 30%	*P*_*2*_	2.87	2.56	2.87	2.74	38.78	32.55 (-16.1%)	38.78 (-0.0%)	35.99 (-7.2%)
	*P*_*3*_	1.86	1.45	1.66	1.57	177.66	84.69 (-52.3%)	152.26 (-14.3%)	118.74 (-33.2%)
	*P*_*4*_	0.15	0.12	0.11	0.11	51.59	18.01 (-65.1%)	35.29 (-31.6%)	26.45 (-48.7%)

() refers to the reduction rates (%) based on the cumulative case counts for “No vaccination”.

Scenario 1 showed the greatest effectiveness in terms of case reduction rate and *R*_t_, primarily because the same level of vaccine coverage was achieved within a shorter and earlier vaccination period. As presented in Table 5, compared with the other scenarios, Scenario 1 consistently produced lower *E*(*R*_t_) regardless of vaccine coverage level or period. In particular, at 70% coverage it achieved an *E*(*R*_t_) of 0.9 in period *P*_*3*_ (below 1) unlike the other scenarios, indicating early and substantial epidemic suppression. Moreover, Scenario 1 also showed clear advantages in reducing cumulative case counts, recording 26.29, 33.6 and 4.43 cumulative cases in periods *P*_*2*_–*P*_*4*_, respectively. These correspond to reductions of 33.2%, 81.1%, and 91.4% compared with the no vaccination scenario in terms of the cumulative case counts.

Scenario 2, which commenced approximately two weeks later than Scenario 1 and was implemented over a longer duration, demonstrated reduced effectiveness. In terms of cumulative case counts, Scenario 2 recorded 38.78, 126.36, 21.3 cases in period *P*_*2*_, *P*_*3*_, and *P*_*4*_, respectively, corresponding to reduction rates of 0%, 28.9%, and 58.7%. Due to the absence of vaccination during *P*_*2*_, the reductions observed in *P*_*3*_ and *P*_*4*_ were smaller than those in Scenario 1, despite vaccination being implemented during these periods.

In Scenario 3, although the initiation timing mirrored that of Scenario 1, the extended vaccination period (by approximately three weeks) resulted in diminished impact. The cumulative case counts were 32.7, 71.83, 10.86 in *P*_*2*_, *P*_*3*_, and *P*_*4*_, respectively, corresponding to reduction rates of 15.7%, 59.6%, and 78.9%. These reductions were greater than those observed in Scenario 2 but consistently lower than those achieved in Scenario 1, primarily due to the slower pace of vaccination. These findings underscore the critical role of both the initiation timing and duration of vaccination campaigns in determining overall effectiveness.

**Fig 5:**
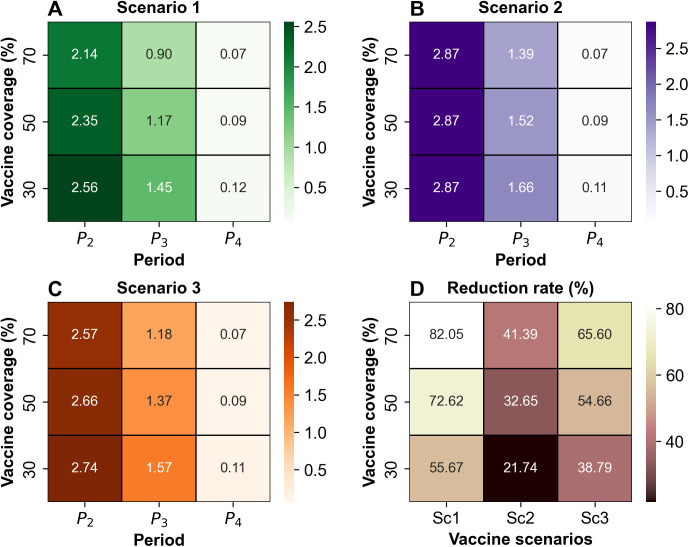
Simulation results for comprehensive vaccination strategies. Summary of the overall results for effective reproduction number (*E*(*R*_t_)) and case reduction rate using heatmaps. A., B., and C. summarize the *E*(*R*_t_) values by period and vaccine coverage for scenarios 1, 2, and 3, respectively. D. visualizes the case reduction rate based on vaccine coverage for Scenario 1 (Sc1), Scenario 2 (Sc2), and Scenario 3 (Sc3).

Fig 5 illustrates the *R*_t_ and case reduction rates for each vaccine scenario, summarized as heatmaps. In the heatmaps, the horizontal axis represents vaccine coverage levels, while the vertical axis indicates either the time period or the vaccine scenarios. Here, we comprehensively evaluated our vaccine scenarios through two key metrics. We set the ORI scenarios according to three criteria: vaccine coverage, the timing of vaccination, and the duration of the vaccination campaign. Our analysis compared the differences in the reduction rate across all period under various vaccine scenarios, and the results are summarized in Fig 5D. A clear hierarchy of impact among the key factors was observed. We found that the timing of vaccine initiation had the largest impact, with differences of up to 40.66%. This was followed by vaccination campaign duration, which showed a maximum difference of 17.96%. Lastly, vaccine coverage accounted for a maximum difference of 16.95%. Based on these findings, it is clear that early and rapid vaccination yields the greatest effectiveness. Scenario 1, which involved the earliest and most concentrated vaccination period, consistently achieved the lowest *E*(*R*_t_) and the highest case reduction rate. This highlights the importance of not only high vaccine coverage but also the timing and speed of deployment in maximizing public health benefits.

### 3.2. Cost-effectiveness analysis

The analysis illustrated in Table 6 presents the health and economic outcomes of three typhoid vaccination scenarios compared to a no-vaccination baseline. Each scenario explores three levels of vaccine coverage, 70%, 50%, and 30%, providing a comparative framework to assess the impact of coverage intensity and timing on disease outcomes and cost-effectiveness. The results consistently show that increasing vaccine coverage yields greater public health benefits, including higher numbers of typhoid cases and deaths averted, alongside more favorable cost-effectiveness metrics.

**Table 6:** Health and economic outcomes of typhoid vaccination scenarios compared to no vaccination.

	Health outcomes				Economic outcomes			
	Cumulative cases	Cases averted	Total typhoid- related deaths	Typhoid- related deaths averted	Total cost (US$)	DALYs averted	ICER per DALY averted (US$)	ICER per case averted (US$)
No vaccination	8741	–	219	–	0	–	–	–
**Scenario 1: Early**								
*κ *= 70%	1569	7172	39	180	3,464,800	7469	464 (HCE)	483 (HCE)
*κ *= 50%	2393	6348	60	159	2,503,100	6610	379 (HCE)	394 (HCE)
*κ *= 30%	3875	4866	97	122	1,556,000	5068	307 (HCE)	320 (HCE)
**Scenario 2: Late**								
*κ *= 70%	5123	3618	128	91	3,543,700	3768	941 (CE)	979 (CE)
*κ *= 50%	5888	2853	147	72	2,580,700	2972	868 (CE)	904 (CE)
*κ *= 30%	6841	1900	171	48	1,621,900	1979	820 (CE)	854 (CE)
**Scenario 3: Combined**								
*κ *= 70%	3007	5734	97	142	3,496,700	5972	586 (HCE)	610 (HCE)
*κ *= 50%	3963	4778	99	120	2,538,000	4975	510 (HCE)	531 (HCE)
*κ *= 30%	5351	3390	134	85	1,588,800	3531	450 (HCE)	469 (HCE)

HCE: highly cost-effective (≤US$656.6), CE: cost-effective (≤US$1969.8), *κ*: vaccine coverage, DALY: disability-adjusted life-year, ICER: incremental cost-effectiveness ratio.

Scenario 1, in which vaccination occurred early during *P*_*2*_ (February 10–February 24, 2015), demonstrated the most substantial health impact across all coverage levels. At 70% coverage, Scenario 1 averted 7,172 cases and 180 deaths, outperforming other scenarios. Even at lower coverage levels (50% and 30%), Scenario 1 continued to yield significant reductions in both morbidity and mortality. Economically, it was the most efficient strategy, with the lowest ICERs: US$464 per DALY averted and US$483 per case averted at 70% coverage, both within the “highly cost-effective” threshold (≤US$656.6 per DALY). These findings suggest that early implementation of vaccination, even over a shorter period, can yield optimal outcomes when paired with high coverage.

In contrast, Scenario 2, which delayed vaccination until *P*_*3*_ (February 24–March 17, 2015), resulted in the poorest performance in terms of cases and deaths averted. At 70% coverage, Scenario 2 averted only 3,618 cases and 91 deaths, approximately half the impact of Scenario 1. ICERs were also higher, at US$941 per DALY and US$979 per case, placing the strategy within the cost-effective range (≤US$1969.8) but not highly cost-effective. The diminished impact of Scenario 2 highlights the importance of early vaccination timing, particularly during the acceleration phase of an outbreak when rapid transmission can be curtailed through timely immunization.

Scenario 3 offered a middle-ground approach by extending vaccination efforts across both *P*_*2*_ and *P*_*3*_ (February 10–March 17, 2015). At 70% coverage, it averted 5,734 cases and 142 deaths, more than Scenario 2, but fewer than Scenario 1. From a cost perspective, it remained highly cost-effective, with an ICER of US$586 per DALY and US$610 per case. These findings suggest that while a prolonged vaccination period can improve outcomes over a delayed start, early concentration of efforts (as in Scenario 1) remains superior when both effectiveness and cost are considered.

Across all scenarios, coverage level significantly influenced outcomes. Increasing vaccine coverage from 30% to 70% consistently improved health outcomes and enhanced cost-effectiveness. For instance, within Scenario 1, moving from 30% to 70% coverage increased the number of averted cases from 4,866–7,172, and averted deaths from 122 to 180, while maintaining favorable ICERs. This trend underscores the value of achieving higher vaccine uptake in maximizing the public health impact of typhoid immunization programs.

In summary, the combination of early timing (Scenario 1) and high coverage (70%) provides the most efficient and effective strategy for typhoid control. It maximizes the number of averted cases and deaths while minimizing costs per health outcome. Although delayed (Scenario 2) or prolonged (Scenario 3) interventions still offer benefits, they do not achieve the same level of impact as early, high-coverage strategies. These findings highlight the critical role of swift public health response and adequate vaccination scale in managing typhoid outbreaks cost-effectively.

## 4. Discussion

This study evaluated the health and economic impacts of deploying typhoid conjugate vaccines as part of outbreak response immunization strategies, using a dynamic compartmental model calibrated to the 2015 typhoid outbreak in Kampala, Uganda. Our findings reinforce the importance of timely vaccination, with early, high-coverage campaigns leading to the most substantial reductions in cases, deaths, and disability-adjusted life years. These strategies also proved to be highly cost-effective based on WHO thresholds, particularly when implemented early in the outbreak.

Our results align with previous studies that emphasize the efficacy of TCVs in endemic settings [28,29,31,33]. However, unlike earlier models focusing primarily on routine immunization, this study uniquely highlights the potential of TCVs in reactive outbreak contexts. The model shows that early intervention significantly reduces the effective reproduction number (*R*_t_), helping to suppress transmission during critical stages of an outbreak. As demonstrated in Scenario 1, an *E*(*R*_t_) below 1 can be achieved, which is crucial to halting epidemic spread during *P*_*3*_.

Among the three vaccination scenarios tested, the timing of implementation had the greatest influence on outcomes, surpassing even campaign duration and coverage in determining overall effectiveness. This underscores the importance of robust surveillance systems capable of early outbreak detection and prompt vaccine deployment. Without timely action, the benefits of even high-coverage campaigns are diminished, as seen in the reduced impact of late-starting strategies.

Economically, our analysis shows that early vaccination campaigns are not only effective but also highly cost-efficient. The lowest ICER observed was US$464 per DALY averted, categorizing the strategy as highly cost-effective by WHO standards. These findings are consistent with prior cost-effectiveness studies that have demonstrated TCVs to be economically favorable in both routine and outbreak contexts [28,31,32].

The model’s reliance on real outbreak data provides a valuable, context-specific framework for evaluating vaccine response strategies. This is particularly important for countries like Uganda that experience recurrent typhoid outbreaks but lack the resources for broad routine immunization coverage. By using retrospective data and counterfactual simulations, our approach provides actionable insights for policymakers considering the introduction or expansion of TCV programs.

Our findings have several important implications for public health decision-makers, particularly in LMICs where typhoid fever outbreaks are more frequent due to limited access to safe water and sanitation. One critical takeaway is the importance of rapid vaccine deployment to achieve significant public health benefits. Given the practical challenges faced in these settings, such as weak surveillance systems and logistical difficulties in vaccine procurement and distribution, our results suggest that strengthening public health infrastructure is important to ensuring timely vaccination during outbreaks. Improving disease surveillance systems to reduce the time between pathogen circulation and outbreak detection could lead to faster vaccine deployment. In addition, streamlining processes for vaccine requests and delivery would be essential for optimizing the impact of ORI programs. In this context, our findings emphasize the broader need for investments in public health infrastructure, not only for typhoid but also for other infectious diseases that disproportionately affect LMICs.

There are limitations to our approach. One notable limitation is the absence of age stratification in our model. The data were not stratified by age group, which limits our capacity to test the impact of age-specific vaccination strategies. Children under 15 years of age account for a significant proportion of typhoid cases in endemic areas [8], future models could benefit from incorporating age-specific vaccination strategies. Secondly, while our model assumes a fixed reporting rate for symptomatic cases due to the lack of data, the true rate may vary significantly across regions and over time, introducing uncertainty into the model’s projections. Further research could address these uncertainties through the collection of more granular data on reporting rates. Thirdly, there is uncertainty in the population immunity at the beginning of the outbreak. Population immunity influences the estimates of the transmission rates, which will in turn significantly influence the impact of the ORI. However, our estimates of transmission rates for four times intervals appear to be consistent with the estimates from other studies of other settings [27,54] which support our estimate of the population immunity. Fourthly, while the assumption regarding the *γ* value for progression to chronic carriage has limitations, the use of a literature-informed *γ* in calculating *R*_0_ produced results that aligned well with observed trends. Finally, our analyses are based on a single outbreak (the 2015 Kampala typhoid outbreak). While this provides valuable insights into the potential health and economic impacts of outbreak response immunization (ORI), it raises questions about the generalizability of our findings. Typhoid outbreaks vary in transmission dynamics, population structure, healthcare infrastructure, and sociobehavioral factors, all of which can influence the effectiveness and cost-effectiveness of vaccination strategies. For example, several historic outbreaks (e.g., Kasese, Uganda [53,55] and Lusaka, Zambia [56]) were sustained for longer durations than the Kampala outbreak modeled here. In such settings, delayed ORI may still yield substantial benefits, beyond what our Kampala-based simulations capture. Thus, while our qualitative insight that timeliness is critical remains robust, caution is warranted in extrapolating the quantitative results to other contexts. Future studies should validate these findings using data from multiple outbreaks across diverse epidemiological settings.

Despite these limitations, the study highlights critical operational factors that determine the success of ORI strategies. These include the availability of vaccine stockpiles, coordination of distribution logistics, and efficient communication with at-risk populations. Investment in these areas is essential to ensure the rapid rollout of TCVs during future outbreaks.

In conclusion, this study demonstrates that early, high-coverage deployment of TCVs in response to typhoid outbreaks is both effective and cost-saving. Mathematical modeling based on outbreak data offers a valuable tool for optimizing vaccination strategies and informing public health policy. As countries aim to strengthen their outbreak preparedness, our findings support the integration of TCVs into emergency response plans as a key intervention for reducing typhoid burden and improving health outcomes.

## Supporting information

S1 Appendix Supplementary material. Supplementary material associated with this article can be found in the online version.
(DOCX)
