# Supplementary material for: Effective strategies for typhoid conjugate vaccine delivery: Health and economic insights from the 2015 Kampala outbreak
Source: PLoS Negl Trop Dis. 2025 Oct 7;19(10):e0013566. doi: 10.1371/journal.pntd.0013566 (PMC12527134; doi:10.1371/journal.pntd.0013566)
Supplement: S1 Appendix — Supplementary material associated with this article can be found in the online version. (DOCX) [file pntd.0013566.s001.docx]

**Supplementary material**

Yeonsu Lee^a,§s^, Pamela Kim N. Salonga^a,§^, Changdae Son^a^, Geunsoo Jang^b^, Dae-Hyup Koh^c^, Jong-Hoon Kim^c,*^*,* Hyojung Lee^a,*^

^a^Department of Statistics, Kyungpook National University, Daegu, 41566 Republic of Korea

^b^School of Computing and Augmented Intelligence, Arizona State University, Tempe, AZ, USA

^c^Epidemiology, Public Health, Impact, International Vaccine Institute, Seoul, 08826 Republic of Korea

1. **Derivation of the basic reproduction number (**$\boldsymbol{R}_{\boldsymbol{0}}$**) and effective reproduction number (**$\boldsymbol{R}_{\mathbf{t}}$**)**

The basic reproduction number ($R_{0}$) and the effective reproduction number ($R_{t}$) were derived using the next generation matrix framework [1]. The basic reproduction number $R_{0}$ assumes a fully susceptible population, meaning that $S(t)=N(t)$. In contrast, the effective reproduction number $R_{t}$ accounts for the reduction in the number of susceptible individuals over time. The derivation process is detailed below.

In our model, the infectious compartments are $I(t)$ and $C(t)$, where $I(t)$ represents infectious individuals and $C(t)$ represents chronic carriers. Vaccination terms were not included during the derivation of the reproduction numbers, as vaccination campaigns were not implemented during the period covered by the observed data. Accordingly, the dynamics of the infectious compartments without vaccination are governed by the following equations:

$$\lambda(t)=\frac{\beta(t)}{N(t)}(I(t)+\gamma C(t))$$

$$\frac{dI(t)}{dt}=\lambda(t)S(t)-\delta I(t)+\mu I(t)$$

$$\frac{dC(t)}{dt}=\delta\theta I(t)-\mu C(t)$$

Based on the dynamics described above, the transmission matrix $F$ and the transition matrix $V$ are constructed. The transmission matrix $F$ represents the appearance of new infections, while the transition matrix $V$ accounts for transitions out of the infectious compartments. These matrices are obtained by taking partial derivatives of the new infection and transition terms with respect to the infectious compartments, and can be written as:

$$F= \left[ \begin{matrix} \frac{\partial F_{1}}{\partial I} & \frac{\partial F_{1}}{\partial C} \\ \frac{\partial F_{2}}{\partial I} & \frac{\partial F_{2}}{\partial C} \end{matrix} \right]=\left[ \begin{matrix} \frac{\beta\left( t \right)}{N\left( t \right)}S(t) & \frac{\beta\left( t \right)\gamma}{N\left( t \right)}S(t) \\ 0 & 0 \end{matrix} \right]=\left[ \begin{matrix} \beta(t) & \beta(t)\gamma\\ 0 & 0 \end{matrix} \right]$$

$$V= \left[ \begin{matrix} \frac{\partial V_{1}}{\partial I} & \frac{\partial V_{1}}{\partial C} \\ \frac{\partial V_{2}}{\partial I} & \frac{\partial V_{2}}{\partial C} \end{matrix} \right]=\left[ \begin{matrix} \delta+\mu& 0 \\ -\delta\theta& \mu\end{matrix} \right]$$

Using the matrices $F$ and $V$, the next generation matrix $K$ is given by

$$K=FV^{-1}=\frac{1}{\mu(\delta+\mu)}\left[ \begin{matrix} \frac{\beta(t)}{\delta+\mu}+\frac{\delta\theta\beta(t)\gamma}{(\delta+\mu)\mu} & \frac{\beta(t)\gamma}{\mu} \\ 0 & 0 \end{matrix} \right]$$

In the next generation matrix $K$, the dominant eigenvalue corresponds to the basic reproduction number $R_{0}$, which is given by

$$R_{0}=\frac{\beta(t)(\mu+\delta\theta\gamma)}{\mu+\delta\theta\gamma}$$

The effective reproduction number $R_{t}$ was calculated by adjusting $R_{0}$ according to the proportion of susceptible individuals:

$$R_{t}=R_{0}\times\frac{S(t)}{N(t)}$$

where $S(t)$ and $N(t)$ denote the number of susceptible individuals and the total population size at time $t$, respectively.

**Table A.** **Initial condition of typhoid modeling.**

| **Compartment** | **Definition** | **Initial value** |
| --- | --- | --- |
| $N_{0}$ | The number of individuals in the population | 1,400,000 |
| $S_{0}$ | Initial number of susceptible individuals | $N_{0}$-$I_{0}$ |
| $I_{0}$ | Initial number of infectious individuals | 1 |
| $R_{0}$ | Initial number of recovered individuals with temporary immunity | 0 |
| $C_{0}$ | Initial number of chronic carriers | 0 |
| $V_{0}$ | Initial number of vaccinated individuals | 0 |

$N_{0}$: the population of Kampala as reported by Kabwama et al [2].

**References**

1. Van den Driessche P, Watmough J. Reproduction numbers and sub-threshold endemic equilibria for compartmental models of disease transmission. Mathematical Biosciences. 2002;180(1-2):29-48. doi:10.1016/S0025-5564(02)00108-6
2. Kabwama SN, Bulage L, Nsubuga F, et al. A large and persistent outbreak of typhoid fever caused by consuming contaminated water and street-vended beverages: Kampala, Uganda, January – June 2015. BMC Public Health. 2017;17(1). doi:10.1186/s12889-016-4002-0
